# Texture and Neonicotinoid Exposure Shape Bacterial Assemblages and Functions in Agricultural Soils: Responses Over Prolonged Exposure

**DOI:** 10.1111/1758-2229.70395

**Published:** 2026-08-03

**Authors:** Sharmin Akter, Julia Jasonsmith, Nilantha R. Hulugalle, Craig L. Strong, James O. Latimer

**Affiliations:** ^1^ Fenner School of Environment and Society, College of Systems and Society, Australian National University Canberra Australian Capital Territory Australia; ^2^ Soil Resource Development Institute Ministry of Agriculture Dhaka Bangladesh

**Keywords:** agriculture, bacterial diversity, bacterial network, imidacloprid, neonicotinoids, pesticides, vertisol

## Abstract

The physical and chemical properties of soil fundamentally shape its microbial communities. In a controlled 28‐day microcosm experiment, we assessed bacterial community responses to imidacloprid in three soils with differing textures and classifications: a loamy sand (11 g/100 g clay; red Luvisol), a sandy loam (16 g/100 g clay; red Luvisol) and a clay soil (56 g/100 g clay, Vertisol). Analyses included 16S rRNA gene amplicon sequencing, indicator species analysis, co‐occurrence network analysis and PICRUSt2‐based functional prediction. Imidacloprid exposure elicited soil‐specific shifts in bacterial community structure, primarily altering evenness rather than richness; however, overall diversity patterns were more strongly governed by soil texture and sampling time. Indicator species analysis identified distinct sensitive and tolerant taxa in each soil texture, with a core set of taxa remaining largely unchanged. Co‐occurrence network analysis showed decreased network complexity and increased modularity under imidacloprid, particularly in loamy sand and clay soils, suggesting altered bacterial interaction patterns. Predicted functional profiles showed upregulation of stress‐response pathways and downregulation of energy/nutrient metabolism pathways, implying a community‐level shift toward stress adaptation. Although taxonomic richness remained relatively stable, these reorganisations of community interactions and functional potential suggest changes in bacterial resilience and biogeochemical cycling, which may have implications for long‐term soil health.

## Graphical abstract

In a 28‐day soil microcosm experiment, bacterial communities responded differently to imidacloprid across contrasting soil textures, with soil‐dependent changes in community structure, interaction networks and functional pathways, while overall diversity remained largely stable.

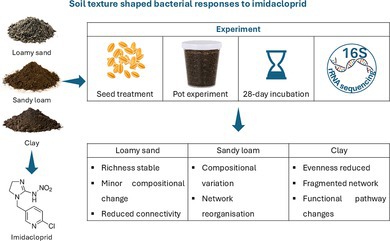

## 1 Introduction

Soil is a complex, heterogeneous medium typically comprised of inorganic mineral particles, organic material, water and air, which together support diverse microbial life critical for agricultural productivity (Buscot 2005). The relative proportions of sand‐, silt‐ and clay‐sized particles dictate a soil's texture. This texture is a fundamental determinant of the soil's physical properties, including moisture, porosity, strength and nutrient availability (Fernandez‐Illescas et al. 2001; Zhu et al. 2021; Lynch et al. 2021). Furthermore, soil texture also governs microbial access to resources and habitat stability through its effects on pore connectivity and substrate availability (Patel et al. 2021; Kravchenko and Guber 2017). Fine‐textured soils rich in clay provide higher water retention but can limit gas exchange and create microenvironments with reduced oxygen levels (Ben‐Noah and Friedman 2016; Al‐Kaisi et al. 2017). These conditions often select for anaerobic or facultative microbial populations, influencing community composition (Tecon and Or 2017). In addition, swelling clays are prone to shrinkage and cracking during drying cycles, which can alter pore connectivity and create preferential flow paths (Qi et al. 2022; Luo et al. 2023). These structural changes may also lead to localised variation in soil physical and chemical properties, influencing microbial habitat stability (Weisskopf et al. 2010; Hullugalle et al. 2001). In contrast, sandy soils with larger pores and lower water‐holding capacity, promote aerobic microbial activity but can expose microbes to environmental stressors such as desiccation (Yang and van Elsas 2018). The distinct physical and chemical environments shaped by soil texture thus create a mosaic of niches, promoting microbial diversity and functional specialisation (Seaton et al. 2020; Naveed et al. 2016; Carson et al. 2010). Texture also dictates how chemicals move through soils and interact with microbial populations. Clays typically exhibit high nutrient retention due to increased surface area adsorption and cation exchange capacity (Wijayawardena et al. 2016; Kumari and Mohan 2021; Sigmund et al. 2022), affecting soil fertility and contaminant mobility (Sigmund et al. 2022). Silty soils provide a similar but reduced, long‐lasting supply of nutrients to microbial communities through slower solute diffusion due to smaller particles and denser pore structure (Matichenkov et al. 2020). In contrast, sandy soils exhibit increased leaching, which results in more transient availability of both nutrients and contaminants (Nunan et al. 2017).

Imidacloprid is the most widely used neonicotinoid insecticide and has been extensively studied in the context of soil microbial responses (Akter et al. 2023; Wang et al. 2023). It has low volatility and bioaccumulation potential, with a propensity to leach through the soil profile due to its weak sorption properties (Fouad and Abdel‐Raheem 2024; Zhang et al. 2018). The mobility of imidacloprid is particularly enhanced in soils with low organic matter, where reduced adsorption increases leaching potential (Bernardino et al. 2021). Its persistence in soil is highly variable, ranging from 40 days to over 2 years depending on physicochemical properties (Broznić and Milin 2013; Sarkar et al. 2001). Bonmatin et al. (2015) detected imidacloprid in 100% of soils sown with treated seeds in the same year and in 97% of soils one to 2 years after application, with accumulation observed in consecutively treated plots. Prolonged exposure to imidacloprid can lead to shifts in microbial diversity (Cycoń and Piotrowska‐Seget 2015b; Castillo‐Díaz et al. 2017), affecting critical microbial functions, including nutrient cycling, nitrogen fixation, and organic matter decomposition (Cycoń and Piotrowska‐Seget 2015a). These disruptions in microbial processes can ultimately influence soil fertility and plant growth, and therefore, sustainable agriculture and ecosystem stability. Understanding the impacts of imidacloprid on soil microbiota, specifically bacteria, across different soil textures over time will provide greater clarity on the broader environmental consequences of neonicotinoids. In this context, soil physicochemical properties play an important role, as they influence the mobility, sorption and persistence of chemicals in soils (Du et al. 2022; Korz and Muñoz 2025), and are likely to affect bacterial responses to neonicotinoids across different soil textures.

In this study we aimed to (i) identify bacterial taxa and community features (indicator taxa, co‐occurrence patterns and predicted functional pathways) associated with imidacloprid exposure across three soil textures, thereby examining how bacterial responses vary among soils, including temporal variation in diversity and community structure, and (ii) identify the relationships between bacterial diversity and key soil properties including pH, electrical conductivity (EC), organic matter (OM), total carbon (C), total nitrogen (N), available phosphorus (P), exchangeable calcium (Ca), exchangeable magnesium (Mg), exchangeable potassium (K), effective cation exchange capacity (ECEC), exchangeable sodium percentage (ESP) and Ca:Mg ratio.

We hypothesised that bacterial responses to imidacloprid would vary across soil textures over time due to differences in physicochemical properties influencing imidacloprid availability and microbial habitat conditions, and that these responses would be reflected in both community structure and functional potentials.

## 2 Materials and Methods

### 2.1 Neonicotinoid and Seed Material

Analytical‐grade standard of imidacloprid (C_9_H_10_ClN_5_O_2_; ≥ 98.0% purity, HPLC) was procured from Sigma–Aldrich Pty Ltd., Australia and stored according to the manufacturer's recommendations (Sigma–Aldrich 2022). Wheat seeds ( *Triticum aestivum* L.) were purchased locally from Canberra, Australia and stored in a cool, dry laboratory environment until use.

### 2.2 Soil Sampling and Preparation

Three soil samples with varying textures were collected from undisturbed, shaded pastures in Canberra (35.28° S, 149.13° E) and Narrabri (30.33° S, 149.78° E), Australia, which have Köppen–Geiger climate classifications of temperate oceanic (Cfb) and hot semi‐arid (BSh), respectively (Beck et al. 2018). All collection sites had no history of pesticide application. Soil samples were collected from the surface (A horizon: ~0.2 m), and initial field hand texturing provided a quick textural assessment to ascertain suitability. The samples were then transported to the Fenner School of Environment and Society laboratory at the Australian National University, Australia, for detailed analysis.

All samples were air‐dried on the laboratory bench for 1 week. As the Canberra soils were weakly aggregated, most aggregates loosened during sieving. In contrast, due to the strong aggregation of the Narrabri high‐clay soil, larger aggregates were mechanically ground using a jaw crusher. Each soil type was then homogenised to create composite samples and sieved through a ≤ 2 mm mesh to remove dried leaves, roots and other debris. Particle size was determined using the modified hydrometer method (Carter and Gregorich 2007) and their textures were determined to be loamy sand, sandy loam and clay (Soil Science Division Staff 2017). The loamy sand and sandy loam soils originated from Canberra and were classified as red Luvisols, while the clay soil from the Narrabri site was classified as a Vertisol (IUSS Working Group WRB 2015).

Thirteen soil physicochemical properties were analysed from composite samples of each soil. These included pH, EC, OM, total C, total N, C:N ratio, available P, exchangeable Ca, exchangeable Mg, exchangeable K, ECEC, ESP and Ca:Mg ratio. The analytical methods used for these measurements are provided in Table S1. A summary of the soil physicochemical and textural properties is presented in Table 2.

### 2.3 Experimental Design

Wheat seeds were oven‐dried at 100°C for 1 h to prevent germination and associated uptake of imidacloprid by emerging seedlings, thereby maintaining consistent distribution of the insecticide within the soil across pots. Germination tests were performed prior to the trial to confirm the efficacy of heat sterilisation.

Forty‐gram samples of soil were dispensed into 30 mL polypropylene pots and compacted for uniformity using a penetrometer set to a pressure of ~294 kPa. Before adding the soil, two 1.5 mm diameter holes were drilled in the sides of the pot to allow targeted sampling of soil in close proximity to the treated seeds at the designated sampling time. The holes were sealed with adhesive tape until sampling.

Wheat seeds were treated with imidacloprid at commercial application rates of 120 mL of 60% active ingredient per 100 kg seeds (Bayer CropScience Australia 2023). Two imidacloprid‐treated seeds were planted in each pot, and initial soil moisture was adjusted to 32 g/100 g on a gravimetric basis using deionised water to provide sufficient moisture for biological processes without causing waterlogging, ensuring uniform conditions across all pots. Moisture was not re‐adjusted during incubation. Following watering, the pots were covered and held in darkness at a constant room temperature of 22°C to maintain stable conditions.

Arranged in a randomised complete block design, each soil type had 15 pots that contained neonicotinoid‐treated seeds and 15 pots with non‐neonicotinoid seeds. Soil sub‐samples were collected at five intervals (Days 3, 7, 14, 21 and 28) with sampling initiated at the same time each day. Control and imidacloprid‐treated soils were sampled concurrently at each time point, allowing treatment effects to be distinguished from temporal changes during incubation. Triplicate samples were included in each treatment and control group at each sampling time.

On each sampling day, six pots per soil type assigned to that time point were removed from the experiment, and ~0.25 g of soil was withdrawn from each pot using a mini spatula through the pre‐drilled holes in the pot walls. Soil samples were immediately processed for DNA extraction. Each pot was sampled once at its assigned time point.

### 2.4 DNA Extraction and Targeted Amplicon Sequencing

Genomic DNA (gDNA) was extracted from soil samples using the DNeasy PowerSoil Pro Kit, following the manufacturer's protocol (Wu 2022). DNA concentrations were determined using a Qubit 4 Fluorometer (Thermo Fisher Scientific), and quality was assessed with a NanoDrop Lite Spectrophotometer. The V1–V3 regions of the 16S rRNA gene were amplified using primers 27f (Lane 1991) and 519r (Lane et al. 1985) and sequenced at the Ramaciotti Centre for Genomics (UNSW, Australia) using the Illumina MiSeq platform with 2 × 300 base pair read lengths.

### 2.5 Bioinformatics Analyses

Illumina‐derived paired‐end FASTQ files were analysed following prior demultiplexing and removal of barcodes, adapters and indices. Primers were removed using Cutadapt (v2.10) (Martin 2011). The paired‐end demultiplexed data were processed with the DADA2 workflow (Callahan et al. 2016), including quality filtering based on truncation lengths and expected error thresholds. Reads were truncated to 275 base pairs for forward and 235 base pairs for reverse with an expected error rate of 2 and 5, respectively. Pseudo‐pooling (‘pool = “pseudo”’) was used for sequence inference after dereplication and error estimation. Reads were merged with a minimum 10 bp overlap followed by removal of chimaeras. A final amplicon sequence variant (ASV) table was obtained, and taxonomy was assigned using version 138.1 of SILVA (McLaren and Callahan 2021).

Across samples, sequencing depths ranged from 1274 to 12,110 reads (Table S2). Observed ASV richness across sequencing depth is illustrated using rarefaction curves in Figure S1. All analyses were performed in R Studio v4.4.2 (R Core Team 2024). The sequence data have been uploaded to the Sequence Read Archive of NCBI (PRJNA1251260).

### 2.6 Functional Prediction

Functional pathway abundance was predicted using the Phylogenetic Investigation of Communities by Reconstruction of Unobserved States (PICRUSt2) pipeline (Douglas et al. 2020). First, study sequences were placed into a reference tree using the ‘place_seqs.py’ tool to assign sequences to a phylogenetic tree. Hidden‐state predictions for 16S rRNA gene copy numbers were made using the ‘hsp.py’ tool. Functional predictions for EC numbers and KEGG orthologs (KOs) were obtained based on marker gene abundances.

Pathway abundances were inferred using the ‘pathway_pipeline.py’ function, which integrates the predicted EC numbers and KOs into bacterial pathway profiles. All pathway predictions were based on the MetaCyc reference database embedded within the PICRUSt2 pipeline. The resulting pathway abundance data were then used for downstream analysis.

### 2.7 Data Processing and Statistical Analyses

The non‐chimeric sequence table and taxonomy table from the DADA2 pipeline were exported along with metadata to create a phyloseq object using v1.48 phyloseq package (McMurdie and Holmes 2013). Filtering steps retained bacterial taxa while removing chloroplast and mitochondrial sequences, along with samples containing no ASVs. A sequence‐derived phylogenetic tree was generated from ASV representative sequences using ‘DECIPHER’ for alignment (Wright 2024) and ‘phangorn’ for tree inference (Schliep 2011) and merged with the object, which was then saved for downstream analysis.

To assess alpha diversity, the Chao1 richness estimator and Pielou's Evenness index were calculated, while phylogenetic diversity was evaluated using Faith's Phylogenetic Diversity (Faith's PD) and Mean Pairwise Distance (MPD). Each diversity measure was modelled using linear mixed‐effects models (LMMs), with treatment, soil texture and time as fixed effects and replicate as a random effect. Analysis of Deviance was performed to assess the significance of these factors, and Dunnett's test was used for pairwise comparisons between control and imidacloprid treatments across different soils and sampling days. Indicator Species Analysis was performed using the ‘multipatt’ function from the indicspecies package (Cáceres and Legendre 2009) to identify taxa that were significantly associated with combinations of soil‐treatment categories. The results were filtered to retain taxa with an IndVal greater than 0.7 and *p* values ≤ 0.05, indicating strong indicator species for specific conditions. Ecological Distance‐Based Redundancy Analysis (dbRDA) was performed to explore the bacterial community composition using the Weighted UniFrac distance matrix, which is based on relative abundances and accounts for differences in sequencing depth. Permutational multivariate analysis of variance (PERMANOVA) was applied to evaluate the statistical significance of the main effects (treatment, soil and day) and their interactions (Anderson 2017). The relationship between bacterial communities and soil properties was explored by correlating the Shannon diversity index with various soil properties. A random forest model was applied to assess the importance of soil properties in explaining the variation in Shannon diversity. To control for potential confounding factors, a Partial Mantel test was performed to evaluate the influence of soil properties on bacterial community structure, while accounting for treatment, soil type and sampling day as covariates. The co‐occurrence network analysis was conducted by aggregating the taxa at the Order level using Spearman's rank correlation. Only correlations meeting thresholds of *p* values ≤ 0.05 and |rho| ≥ 0.8 were considered significant. Network parameters, including transitivity, average path length, modularity and network diameter, were calculated for each treatment and soil type. Differential abundance analysis (DAA) of predicted functional pathway abundance was performed using the ‘edgeR’ method, with significance determined based on Benjamini–Hochberg (BH) false discovery rate (FDR)‐adjusted *p* values. All figures were generated in R using ‘ggplot2’ (Wickham 2016), ‘ggridges’ (Wilke 2024), ‘ggvenn’ (Yan 2023), ‘igraph’ (Csárdi et al. 2025; Csárdi and Nepusz 2006) and ‘vegan’ (Oksanen et al. 2024) along with related packages.

## 3 Results

### 3.1 Diversity Profiles in Different Soil and Imidacloprid

**FIGURE 1:**
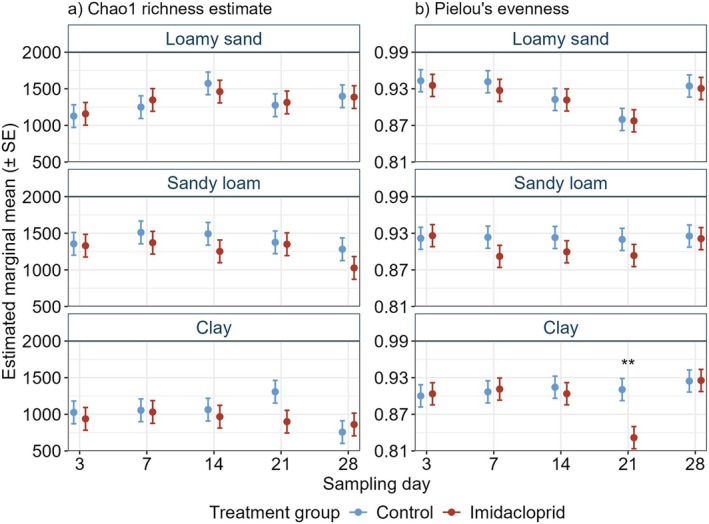
Changes in bacterial alpha diversity across soil textures and treatments over time. (a) Chao1 richness and (b) Pielou's evenness. Points represent estimated marginal means ± SE (*n* = 3).

Analysis of deviance revealed a significant effect of soil on Chao1 richness (Table S3; Figure 1a), indicating that bacterial richness varied across different soil textures. However, Dunnett's test did not detect significant differences between the control and imidacloprid‐treated samples in relation to any soil texture. Contrastingly, Pielou's evenness was significantly influenced by time (Table S4; Figure 1b), suggesting temporal shifts in community distribution. A treatment effect was detected only in clay soil on Day 21, where evenness was reduced under imidacloprid compared with the control (estimate = −0.08, *p* = 0.003). No other treatment‐related differences were observed across soils or time points. Phylogenetic diversity metrics (Faith's PD and MPD) showed limited and inconsistent responses across soils and time, with no clear overall treatment effects. A significant increase in MPD was observed in sandy loam soil on Day 21, indicating a localised shift in phylogenetic structure (Tables S5–S6; Figure S2b).

### 3.2 Changes in Bacterial Community Composition and Structure

#### 3.2.1 Indicator Species Analysis

**FIGURE 2:**
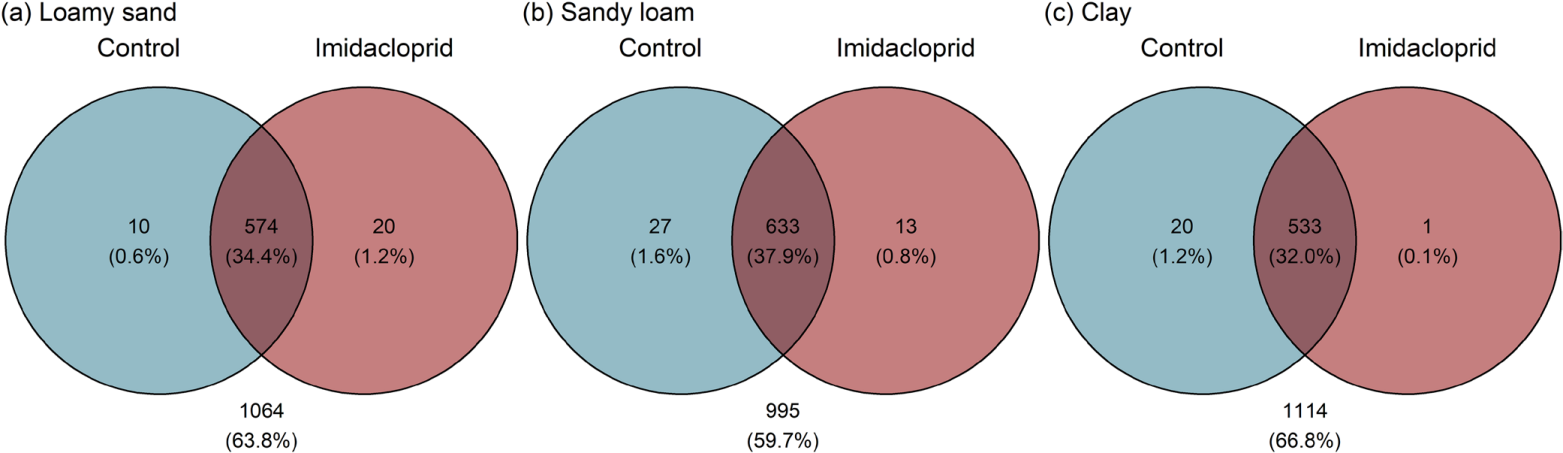
Venn diagrams showing the distribution of indicator taxa between control and imidacloprid at ASV, order, family, genus, and species level across three soil‐texture: (a) Loamy sand, (b) Sandy loam and (c) Clay (IndVal > 0.7; *p* value ≤ 0.05). The blue regions represent taxa unique to the control treatment, the red regions represent taxa unique to the imidacloprid treatment, and the overlapping areas indicate taxa that are significant for both treatments.

A total of 1668 bacterial ASVs were identified through indicator species analysis (IndVal > 0.7, *p* ≤ 0.05; Figure 2). Across soils, most ASVs showed no significant association with either treatment, and many were shared between control and imidacloprid treatments, while only a small proportion were uniquely associated with either group. This pattern varied among soil textures. In loamy sand, 10 ASVs (0.6%) were uniquely associated with the control and 20 (1.2%) with imidacloprid, while in sandy loam, 28 ASVs (1.7%) were unique to the control and 13 (0.8%) to imidacloprid. The strongest imbalance occurred in clay soil, where 20 ASVs (1.2%) were uniquely to the control and only one ASV (0.1%) was uniquely associated with imidacloprid. Most ASVs in clay were either non‐indicators (66.8%) or shared between treatments (32.0%). This pronounced asymmetry indicates that bacterial taxa in clay soil were more strongly associated with the control than with imidacloprid treatment, with very few taxa showing consistent association with the treatment condition. The taxonomic distribution of these indicator species analyses is shown in Table S7.

#### 3.2.2 dbRDA Analysis of Community Structure

**FIGURE 3:**
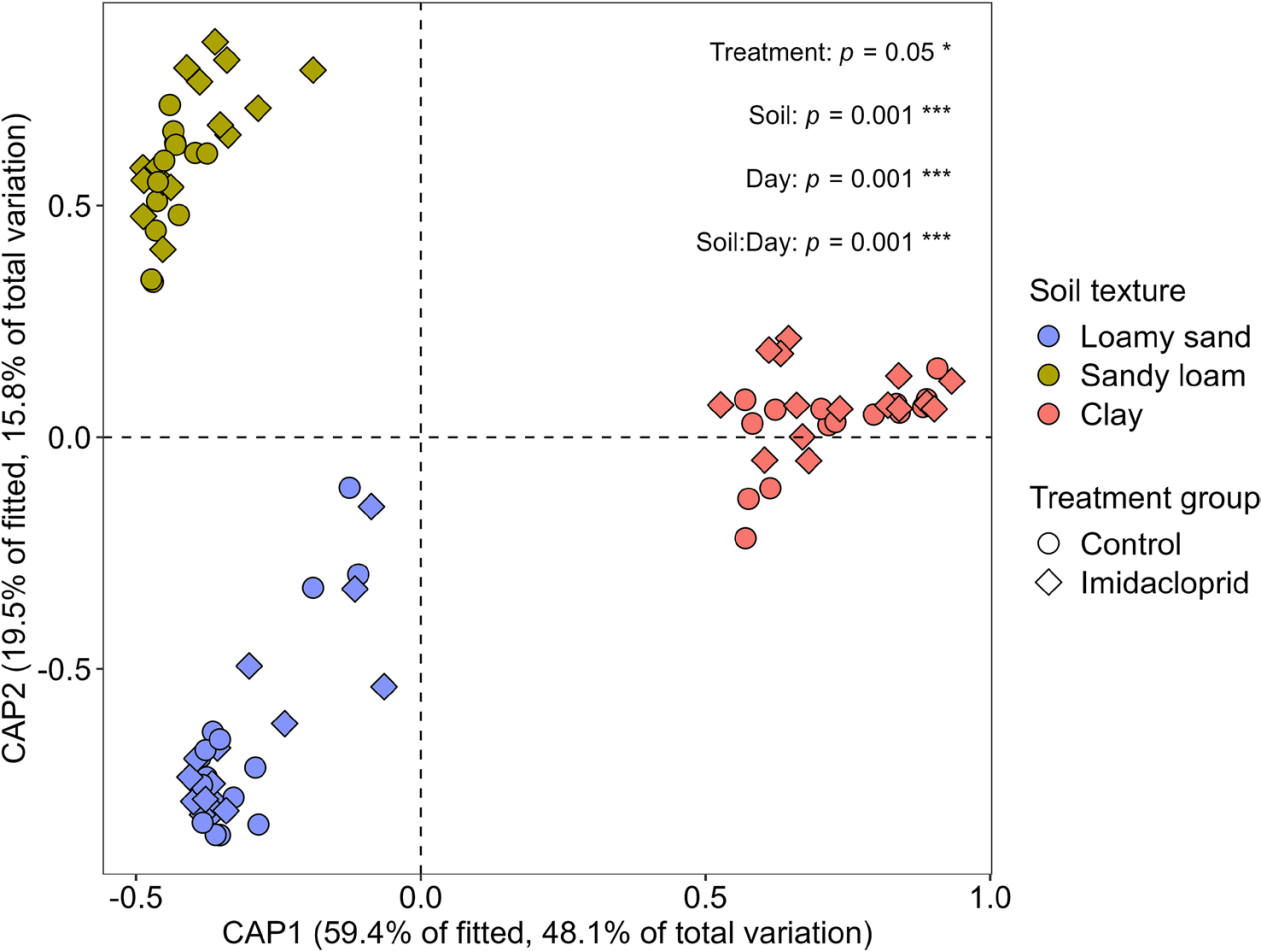
Distance‐based redundancy analysis (dbRDA) plot illustrating bacterial community structure using Weighted UniFrac distances. Colours correspond to different soil textures, while shapes represent the treatment groups. Significance levels are denoted as ***p ≤ 0.001, **p ≤ 0.01, *p ≤ 0.05.

Analysis based on dbRDA scores using PERMANOVA revealed a dominant effect of soil texture on bacterial community structure (*R* ^2^ = 0.623, *p* = 0.001), with additional contributions from the soil × sampling day interaction (*R* ^2^ = 0.082, *p* = 0.001) and sampling day (*R* ^2^ = 0.055, *p* = 0.001), while the treatment effect was small (*R* ^2^ = 0.008, *p* = 0.05). The dbRDA plot (Figure 3) visually supported these findings, showing distinct clustering of bacterial communities by soil texture along the constrained axes. Loamy sand samples were positioned at lower CAP2 values, while sandy loam samples clustered at higher CAP2 values. Clay soils formed a distinct cluster in the positive region of CAP1, with slightly positive CAP2 values, separating them from the other soil types. CAP1 and CAP2 together explained 78.9% of the constrained variation (CAP1: 59.4%, CAP2: 19.5%), corresponding to 48.1% and 15.8% of the total variation, respectively, with additional variation captured by higher‐order axes. Within each soil type, control and imidacloprid‐treated samples largely overlapped, consistent with the small effect of treatment on community structure.

### 3.3 Bacterial Community‐Soil Properties Interaction

Soil physicochemical properties varied across the three soil textures and are summarised in Table 1.

**TABLE 1:** Physicochemical properties of the three soils used in the experiment.

Properties	Loamy sand (Red Luvisol)	Sandy loam (Red Luvisol)	Clay (Vertisol)
pH (1:5 H_2_O)	6.33	6.71	8.74
EC (dS/m) (1:5 H_2_O)	0.069	0.406	0.286
OM (g/100 g)	5.7	8.4	2.8
Total C (g/100 g)	3.3	5.1	1.7
Total N (g/100 g)	0.19	0.43	0.13
C:N ratio	17	11	12
Available P (mg P/kg soil)	35	232	20
Exchangeable Ca (cmol_+_/kg)	6.3	18	25
Exchangeable Mg (cmol_+_/kg)	1.9	3.5	15
Exchangeable K (cmol_+_/kg)	0.44	1.4	1.3
ECEC	8.8	23	44
ESP	1.2	0.16	7.4
Ca:Mg ratio	3.4	5.1	1.7
Sand (g/100 g)	82.08	70.03	28.51
Silt (g/100 g)	6.79	13.94	15.24
Clay (g/100 g)	11.14	16.03	56.25

**FIGURE 4:**
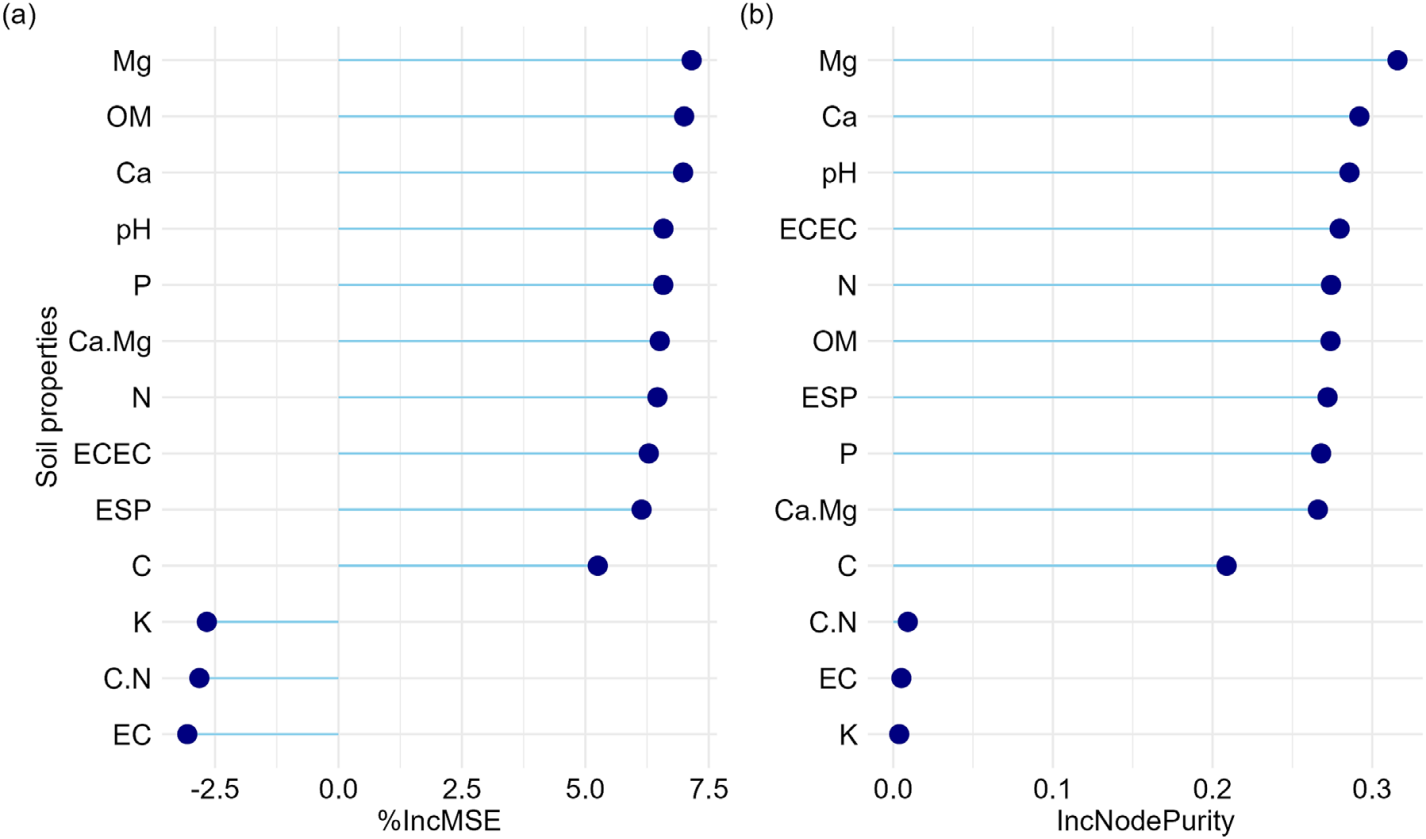
Importance of soil physicochemical properties in predicting bacterial alpha diversity (Shannon index) based on random forest analysis. (a) Shows variable importance ranked by the percent increase in mean squared error (%IncMSE), and (b) shows importance based on increase in node purity (IncNodePurity).

According to the Random Forest model, 16.2% of the variance in Shannon diversity was explained by soil properties, with a mean squared residual of 0.109. Among the 13 soil properties tested, Mg ranked highest, followed by Ca, pH and ECEC, while OM, total N, ESP, available P and Ca:Mg consistently ranked as the most important predictors based on both %IncMSE and IncNodePurity metrics (Figure 4). These properties contributed relatively more to model performance and decision tree structure, indicating that bacterial diversity was associated with a suite of interrelated soil properties rather than a single dominant predictor. In contrast, K, EC and the C:N ratio showed negligible importance, suggesting limited predictive value for Shannon diversity in this system.

**FIGURE 5:**
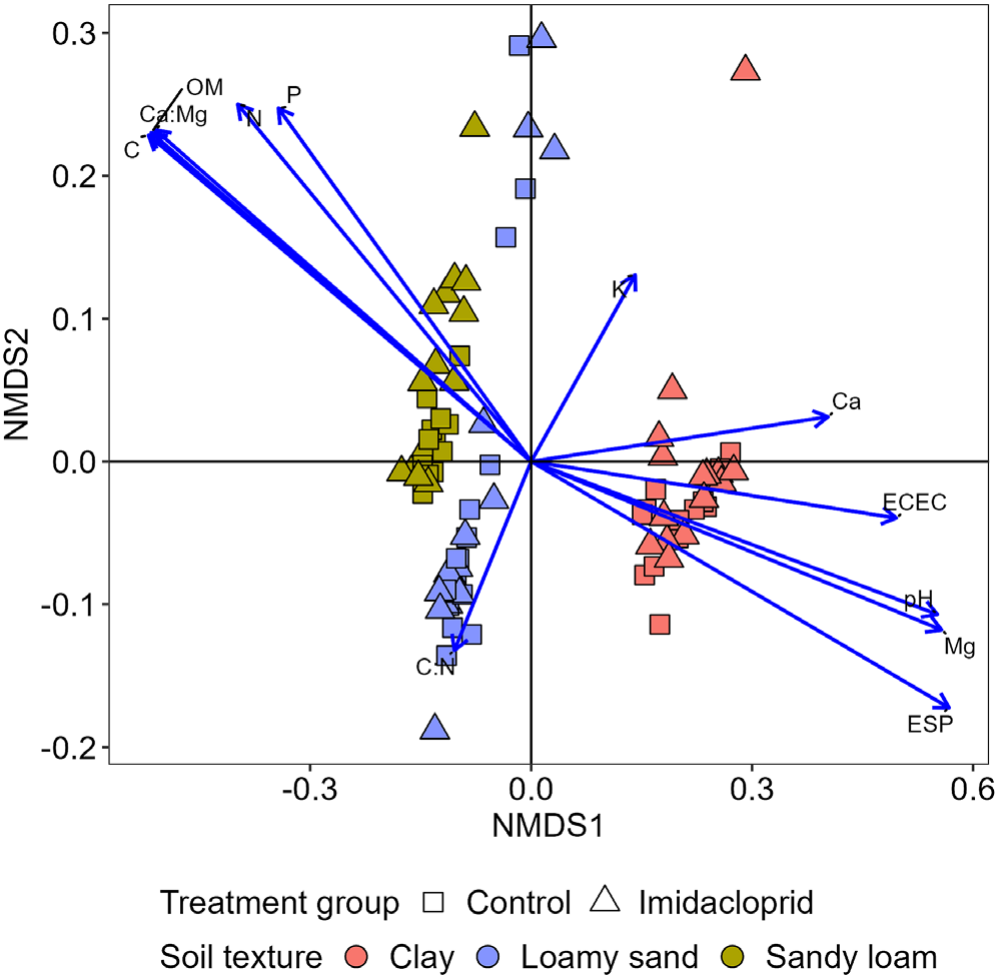
Non‐metric multidimensional scaling (NMDS) ordination of bacterial community structure based on weighted UniFrac distances. Points represent individual samples, coloured by soil texture and shaped by treatment group. Vectors indicate fitted correlations of soil physicochemical properties with community structure; arrow direction shows the gradient, and length reflects the strength of association.

Partial Mantel analysis confirmed a significant relationship between soil physico‐chemical properties and bacterial community structure after controlling for treatment, soil type and sampling day (*r* = 0.36, *p* = 0.001). Vector fitting onto the NMDS ordination (Figure 5) revealed that multiple soil physicochemical properties were differentially associated with bacterial community (*p ≤* 0.032). Strong gradients along NMDS1 were defined by ESP, Mg, pH, OM, C and Ca:Mg (*R* ^2^ ≥ 0.87), while N and P were moderately associated with NMDS2 (*R* ^2^ = 0.61, 0.50, respectively). In contrast, C:N showed a weak association (*R* ^2^ = 0.08). These results indicate that bacterial community variation aligned with multiple, correlated soil physicochemical gradients.

### 3.4 Co‐Occurrence Network Analysis

Bacterial association patterns differed significantly across soil types and treatments. The topological characteristics of the networks were calculated and are summarised in Table 2.

**TABLE 2:** Summary of topological characteristics of co‐occurrence networks across soil textures and treatments.

Soils	Treatments	Nodes	Edges	transitivities	Average path lengths	Modularities	Graph densities	Network diameters	Average degrees	Clustering coefficients
Loamy sand	Control	63	321	0.57	3.04	0.39	0.16	8	10.19	0.65
	Imidacloprid	52	195	0.52	2.28	0.46	0.15	6	7.50	0.61
Sandy loam	Control	53	223	0.46	2.54	0.31	0.16	6	8.42	0.52
	Imidacloprid	54	268	0.49	2.70	0.43	0.19	8	9.93	0.51
Clay	Control	35	94	0.53	1.98	0.44	0.16	4	5.37	0.68
	Imidacloprid	35	57	0.32	3.68	0.63	0.10	9	3.26	0.37

**FIGURE 6:**
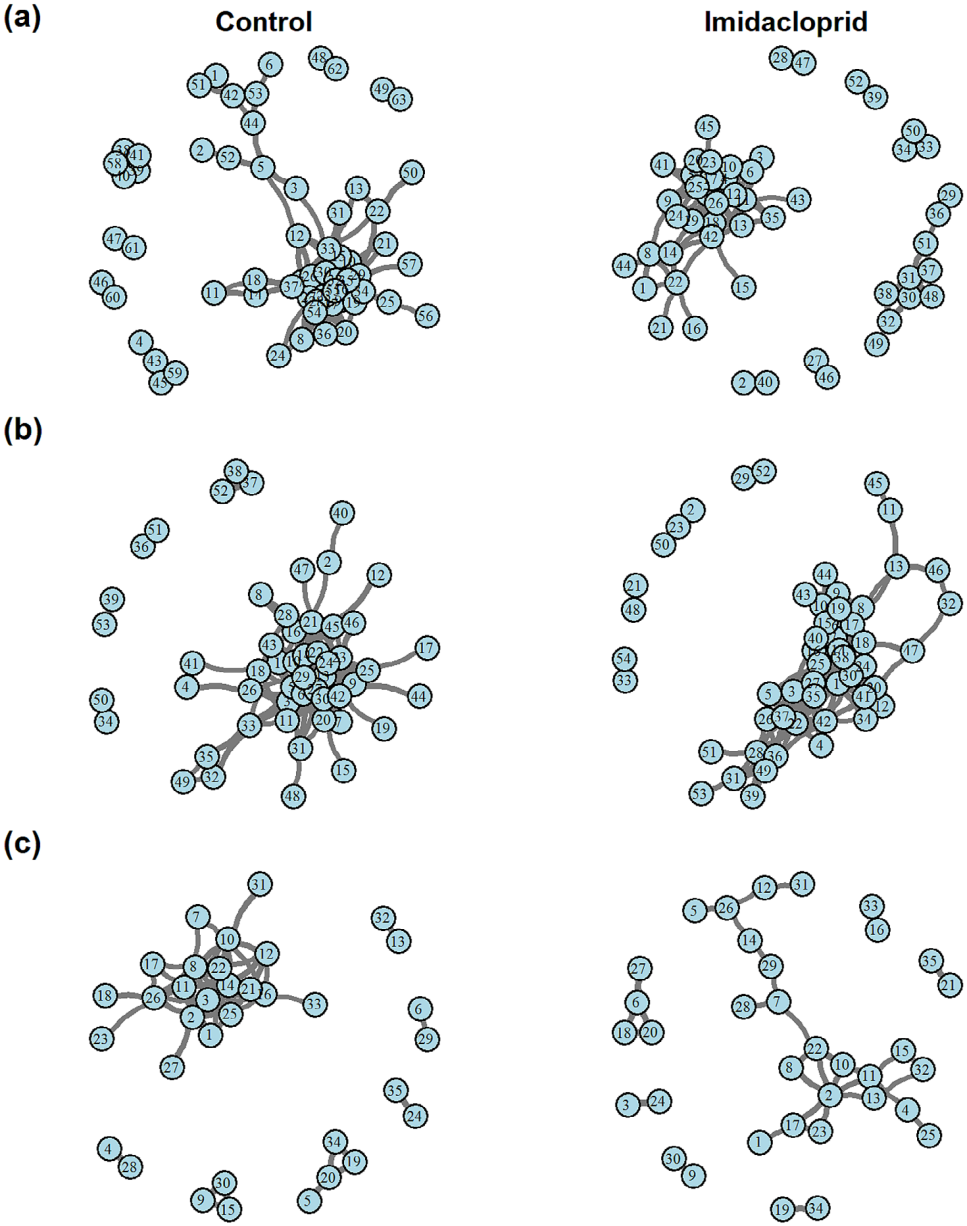
Co‐occurrence networks for bacterial taxa at the order level in different soil types and treatments. (a) Loamy sand, (b) Sandy loam and (c) Clay soil under control and imidacloprid treatments. Nodes represent microbial taxa, and edges represent significant interactions between taxa (|rho| ≥ 0.8, *p* ≤ 0.05).

In loamy sand soil, imidacloprid treatment was associated with a decrease in the number of edges (196 vs. 321 in control) and an increase in modularity (0.47 vs. 0.37), suggesting a less connected and more modular network structure. In sandy loam soil, imidacloprid treatment corresponded to an increase in edges (273 vs. 223 in control) and modularity (0.43 vs. 0.32 in control), suggesting a more connected and more modular network structure. In clay soil, the network under imidacloprid had fewer edges (57 vs. 94 in control) and higher modularity (0.63 vs. 0.44 in control), suggesting a less connected but more modular structure. The network plots for each soil type and treatment are presented in Figure 6, illustrating the network structure and providing a visual representation of the associations between taxa. These visual patterns are consistent with the numerical trends in Table 2, particularly the reduced connectivity and increased modularity, indicative of a more fragmented network structure observed in the clay soil under imidacloprid. The taxon names corresponding to the network nodes for each soil are provided in Tables S8–S10.

### 3.5 Functional Pathway Abundance

**FIGURE 7:**
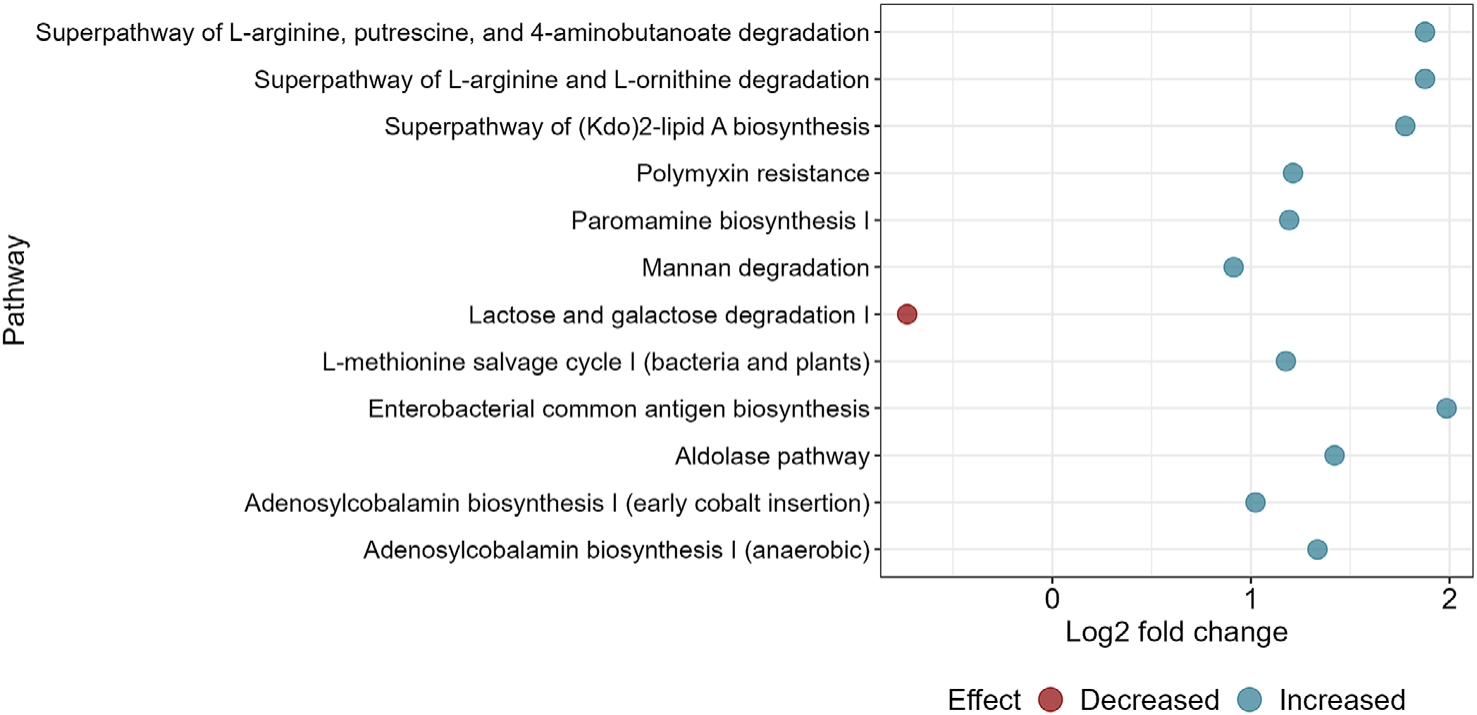
Changes in functional pathway abundance in loamy sand soil. The *x*‐axis shows the log2 fold change (log2FC) in abundance, while the *y*‐axis represents significant (*p*_adj_ ≤ 0.05) individual pathways. Blue points indicate pathways predicted to be more abundant than the control, whereas red points indicate pathways predicted to be less abundant.

The PICRUSt2‐predicted functional pathway analysis identified a total of 427 pathways across the different soil textures. In loamy sand soil, 12 pathways exhibited significant changes (*p*_adj_ ≤ 0.05) under neonicotinoid seed treatment (Figure 7). Key upregulated pathways included enterobacterial common antigen biosynthesis and superpathway of l‐arginine, putrescine and 4‐aminobutanoate degradation, consistent with stress‐related functions. Conversely, lactose and galactose degradation I were significantly decreased, indicating shifts in predicted carbohydrate metabolism.

**FIGURE 8:**
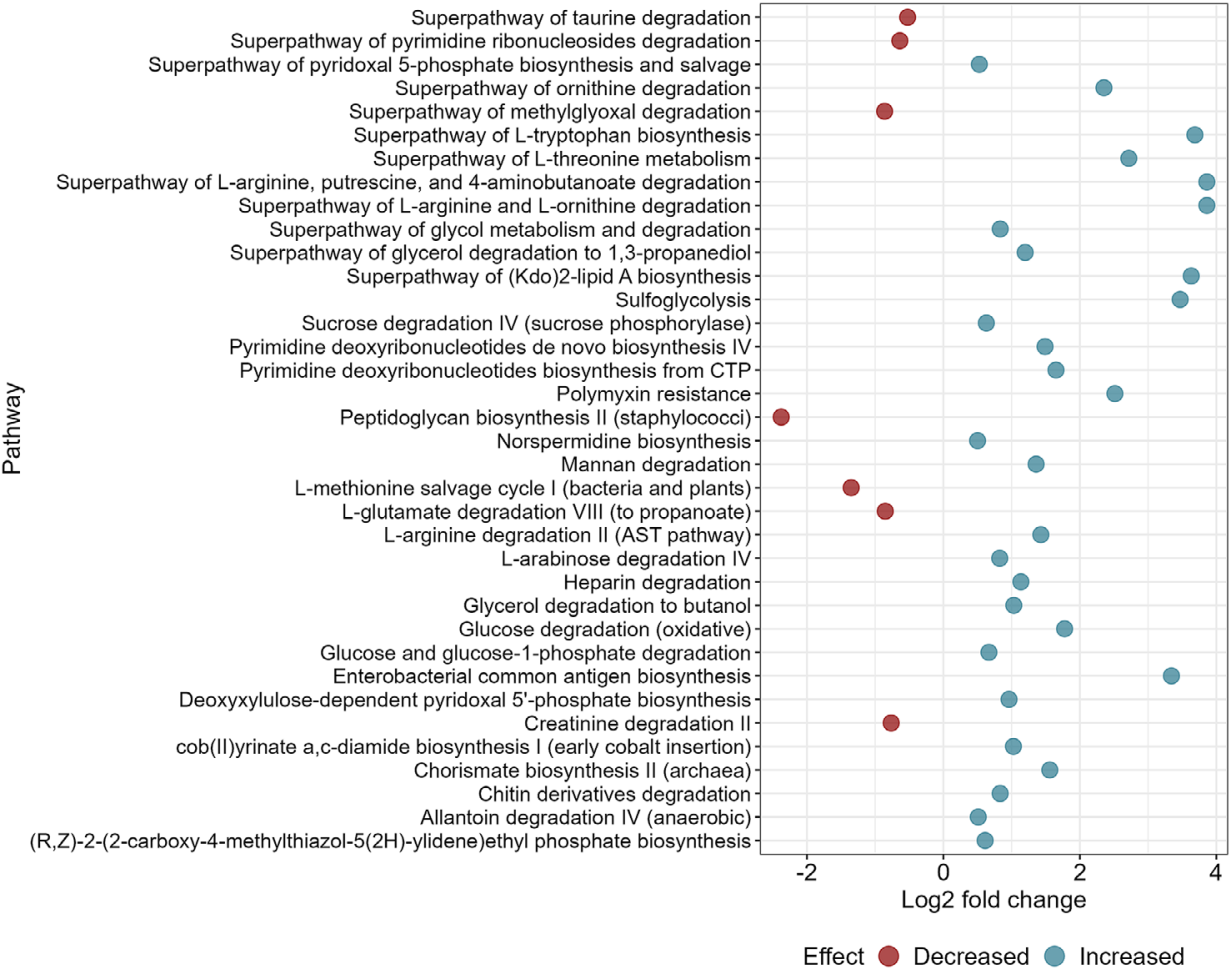
Changes in functional pathway abundance in sandy loam soil. The *x*‐axis shows the log2 fold change (log2FC) in abundance, while the *y*‐axis represents significant (*p*_adj_ ≤ 0.05) individual pathways. Blue points indicate pathways predicted to be more abundant than the control, whereas red points indicate pathways predicted to be less abundant.

In sandy loam soil, 36 pathways showed significant changes (*p*_adj_ ≤ 0.05) under imidacloprid treatment (Figure 8). Prominent upregulations included the superpathway of l‐arginine, putrescine and 4‐aminobutanoate degradation and the superpathway of l‐tryptophan biosynthesis, while the superpathway of both taurine degradation and of pyrimidine ribonucleosides degradation was downregulated, indicating shifts in amino acid and nucleotide metabolism.

**FIGURE 9:**
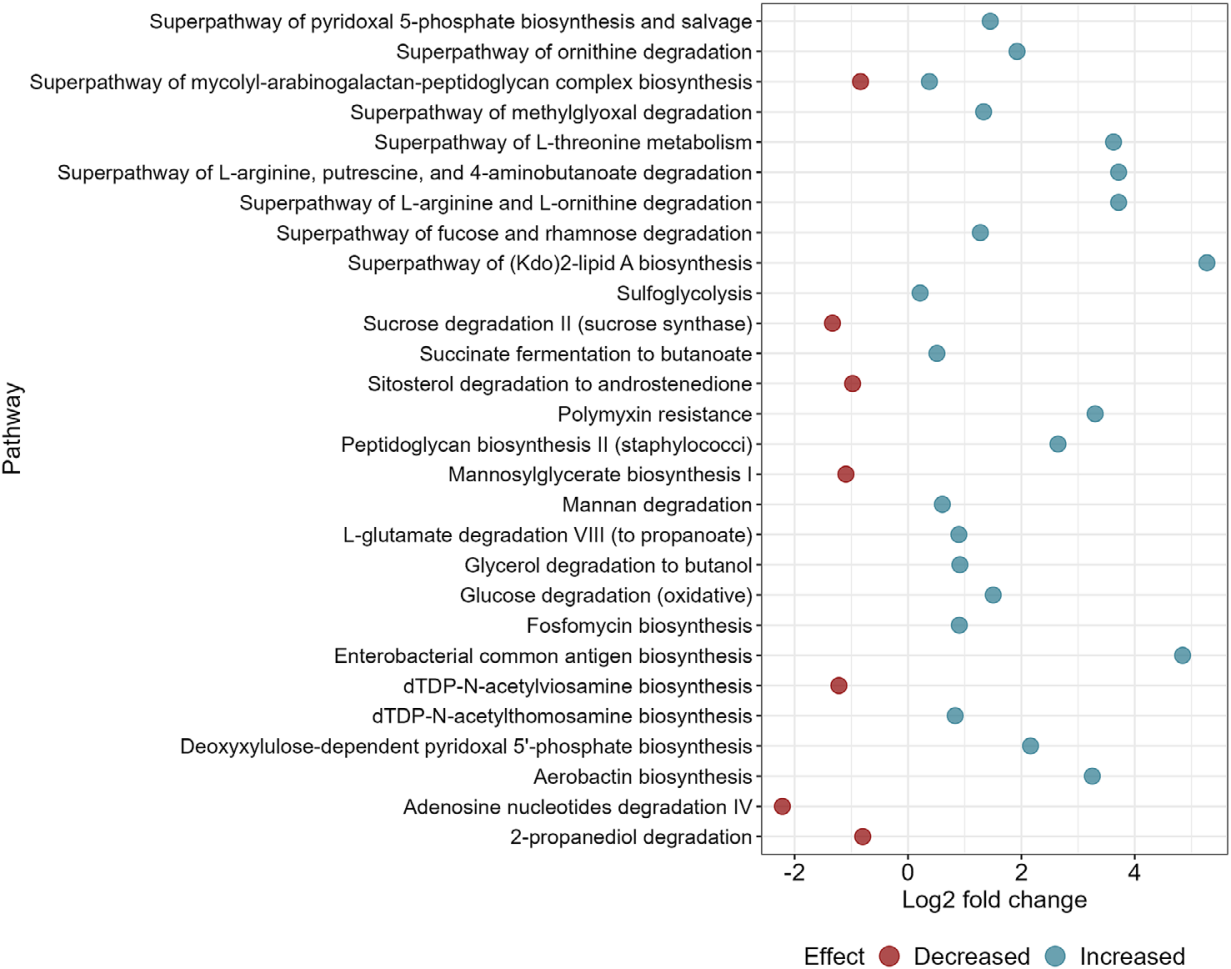
Changes in functional pathway abundance in clay‐textured soil. The *x*‐axis shows the log2 fold change (log2FC) in abundance, while the *y*‐axis represents significant (*p*_adj_ ≤ 0.05) individual pathways. Blue points indicate pathways predicted to be more abundant than the control, whereas red points indicate pathways predicted to be less abundant.

In clay‐textured soil, 29 pathways exhibited significant changes (*p*_adj_ ≤ 0.05) under imidacloprid treatment (Figure 9). Notable upregulation was observed in the superpathway of (Kdo)_2_‐lipid A biosynthesis and enterobacterial common antigen biosynthesis, consistent with stress‐related functions. Several pathways, including adenosine nucleotides degradation IV and sucrose degradation II, were downregulated, indicating changes in predicted energy metabolism.

Overall, across soil types, pathway shifts consistently reflected increased stress‐related functions alongside reductions in carbohydrate, nucleotide and energy metabolism, with more extensive changes in sandy loam and clay soils.

## 4 Discussion

### 4.1 Bacterial Community Dynamics

A reduction in bacterial community evenness was observed only in clay soil on Day 21 (Figure 1b), consistent with previous findings from Parizadeh et al. (2021), who reported significant reductions in bacterial evenness and richness in clay and clay loam soils exposed to thiamethoxam seed treatment. However, in our study, this pattern was not consistently observed across soils or time points. The reduction in evenness may be related to the higher adsorption capacity of clay soils, which may prolong localised microbial exposure to imidacloprid (Muema et al. 2016; Kızılkaya et al. 2004; Masini and Abate 2021; Dankyi et al. 2018). The clay‐textured soil used in this study was a Vertisol, characterised by smectitic minerals and shrink‐swell behaviour, properties that may further influence the retention and spatial distribution of imidacloprid within the soil matrix, potentially contributing to the observed bacterial responses (Muchaonyerwa et al. 2006; Niaz et al. 2023), although these processes were not directly measured in the present study.

Phylogenetic diversity showed limited and soil‐specific responses. An increase in MPD was observed only in sandy loam soil on Day 21, indicating a localised shift in phylogenetic structure (Table S6; Figure S2b). This may reflect reduced competitive dominance or stress‐induced suppression of dominant lineages, allowing the coexistence of phylogenetically diverse, stress‐tolerant taxa (Webb et al. 2002) or increased influence of stochastic processes, such as random colonisation or ecological drift under chemical disturbance (Pérez‐Valera et al. 2015; Chase and Myers 2011). Ecological filtering may also contribute to such patterns depending on trait distribution (Pavoine and Bonsall 2011). However, this increase was not observed at other sampling times or in other soil types.

Indicator species analysis showed that most taxa were shared between the control and imidacloprid treatments, indicating broad overlap in bacterial communities during the 28‐day exposure (Figure 2). Taxa unique to each treatment may represent differential associations under untreated versus neonicotinoid‐exposed conditions. Across all three soils, several taxa were strongly associated with control treatments, notably *Pseudomonas*, *Solirubrobacter*, *Ensifer*, *Marmoricola*, *Rhizobium*, *Microlunatus* and *Chryseolinea*. These taxa are known to play critical roles in nitrogen cycling, phosphorus mobilisation and organic matter degradation, suggesting potential shifts in taxa associated with nutrient‐related functions under imidacloprid exposure (Khoshru et al. 2025; Jara‐Servin et al. 2024; Fox et al. 2007; Potera 2007). This finding aligns with reports of reduced abundance of beneficial rhizosphere bacteria under neonicotinoid exposure (Parizadeh et al. 2021; Gorshkov et al. 2024). These patterns may suggest potential impacts on symbiotic nitrogen fixation and nutrient cycling, as N_2_‐fixing bacteria are particularly sensitive to imidacloprid (Cycoń and Piotrowska‐Seget 2015a). In contrast, several genera were uniquely associated with imidacloprid‐treated soils, including *Mycobacterium*, *Flavobacterium*, *Nakamurella*, *Noviherbaspirillum*, *Rhodococcus* and *Pseudarthrobacter*. These taxa are ecologically equipped to tolerate or metabolise toxic organic compounds like neonicotinoids (Ramírez et al. 2023; Alvarez et al. 2017). In clay soil, the strongest imbalance between control‐ and treatment‐associated taxa was observed, with few taxa uniquely associated with imidacloprid, suggesting a less consistent microbial response to treatment.

The inherent differences among the loamy sand, sandy loam, and clay soil communities along with successional changes over the 28‐day period accounted for substantially more variation in microbial profiles than the presence or absence of the pesticide (Figure 3). This result underscores that the baseline edaphic properties and temporal dynamics of the microcosms outweighed the treatment effect on the microbiome. Such findings are consistent with broader ecological patterns, where soil physico‐chemical properties, particularly texture, exert strong selective pressure on microbial communities, leading to distinct assemblages in sands versus clays, regardless of experimental treatments (Liu et al. 2024). This emphasises the critical role of soil texture in structuring bacterial communities, consistent with previous research that has demonstrated the importance of soil characteristics in paichongding‐treated soils (Cai et al. 2016). Similarly, a study applying thiamethoxam in two soil types, silt loam and silt clay, found that both soil texture and time significantly shaped microbial community structure, with pesticide effects varying across soil types and over time (Wu et al. 2021). Soil texture may also influence imidacloprid behaviour through sorption–desorption processes that differ between sandy and clay‐rich soils, which may contribute to the muted treatment effects observed across soils (Krohn and Hellpointner 2002). In finer‐textured soils (e.g., our vertisol soil), greater sorption of imidacloprid to soil particles, particularly clay surfaces, has been reported (Fouad and Abdel‐Raheem 2024), with organic matter acting as an important sorptive medium for imidacloprid (Didović et al. 2022).

These findings further highlighted that inherent soil properties strongly shape microbial diversity and community structure (Table 1; Figures 4 and 5). High OM and total carbon C create abundant energy sources and microhabitats, often supporting greater bacterial diversity (Tian et al. 2021; Bastida et al. 2021). Likewise, richer N and P availability can promote diverse microbial growth, as soil bacterial diversity and composition are largely driven by total C, N and P stoichiometry (Zhang et al. 2023; Delgado‐Baquerizo et al. 2017). The association of sandy loam soils with nutrient‐related soil properties suggests that nutrient‐enriched soils select for microbial communities adapted to exploit these resources. Consistent with these patterns, clay soils with elevated Ca, Mg, pH, EC and ESP harboured distinct communities tolerant of high base cations (Tang et al. 2019; Kaiser et al. 2016; Zhang et al. 2024). The strong soil physicochemical predictors identified likely reflect underlying environmental gradients associated with substrate availability and habitat heterogeneity, explaining the observed differences in bacterial communities across soil textures.

### 4.2 Bacterial Community Network Structure

Consistent with previous studies, correlation‐based patterns may reflect co‐oscillation of taxa in response to shared environmental conditions rather than direct relationships, and therefore caution is needed in their interpretation (de Vries et al. 2018; Freilich et al. 2018). Imidacloprid exposure induced significant changes in the microbial co‐occurrence network across different soil types. Loamy sand and clay soils showed pronounced network fragmentation, with imidacloprid greatly reducing the number of edges, signifying fewer microbial co‐occurrences, and increasing modularity, leading to more compartmentalised subnetworks. Such fragmentation suggests that many associations were disrupted, isolating microbes into smaller modules, as observed in similar findings where thiamethoxam was applied in clay soil (Zhang, Zhang, et al. 2021). In the clay‐textured soil, lower microbial connectivity under imidacloprid may also be influenced by the inherently discontinuous macropore networks of Vertisols, which are known to limit air diffusion and isolate microbial habitats in swelling clays (Ruan et al. 2021). In sandy loam textured soil, by contrast, imidacloprid increased the number of edges alongside higher modularity, hinting that association patterns were reorganised, even as the network became partitioned into modules. This could reflect selection for stress‐tolerant microbes that associate strongly with each other, thereby compensating for the loss of sensitive species (Li et al. 2022). These network shifts align with broader patterns of microbial responses to agrochemicals, which have been associated with disrupted symbioses and loss of keystone taxa that maintain community structure, which can impair ecosystem functions reliant on microbial interactions (Lv et al. 2025; Meyer et al. 2024; Zhang, Song, et al. 2021).

### 4.3 Functional Pathway Changes Across Soils

The functional profiles presented in this study therefore reflect inferred metabolic potential rather than direct measurement of bacterial activity. Under imidacloprid exposure, each soil type showed a distinct yet convergent shift in microbial functional profiles consistent with stress adaptation. In all soil textures analysed, pathways linked to stress resilience and catabolism of amino compounds were enriched, whereas those for carbohydrate and nucleotide utilisation were repressed. For example, the significant upregulation of polyamine degradation (the l‐arginine/putrescine/4‐aminobutanoate superpathway) suggests activation of a general stress response, consistent with altered polyamine metabolism of microbes under adverse condition (Niu et al. 2024) and previous evidence that polyamine catabolism contributes to bacterial stress responses (Schneider et al. 2013). Likewise, increased biosynthesis of gram‐negative cell envelope components (e.g., enterobacterial common antigen and Kdo_2_‐lipid A) is consistent with potential cell envelope fortification under chemical stress, in line with observations for difenoconazole pesticide pressure, which induced membrane‐associated protective functions (Feng et al. 2025). Conversely, the downregulation of pathways for complex sugar breakdown (e.g., lactose/galactose, sucrose) and nucleotide metabolism indicated metabolic reorganisation under imidacloprid exposure. These patterns aligned with reports that pesticide‐exposed soil communities prioritised stress‐defence and detoxification mechanisms such as contaminant degradation enzymes at the expense of growth‐oriented metabolism (Senabio et al. 2024). While the specific pathways affected varied across loamy sand, sandy loam and clay, the overall functional response was one of community‐level stress adaptation and metabolic reorganisation in the presence of imidacloprid. Ecologically, these functional shifts have profound implications for soil ecosystem processes. Future studies incorporating measurements of soil enzyme activities could further clarify how these predicted functional shifts translate into changes in soil biogeochemical processes, while direct quantification of imidacloprid residues would help to better characterise exposure dynamics and strengthen interpretation of bacterial responses across contrasting soil textures.

## 5 Conclusion

Imidacloprid exposure across loamy sand, sandy loam and clay textured soils induced soil‐texture specific shifts in bacterial communities. Alpha diversity showed a context‐dependent response to imidacloprid, with a reduction in community evenness observed only in clay soil at a single time point, while richness remained unaffected. Soil texture was the primary driver of community composition, with time contributing marginally. Indicator species analysis revealed that many bacterial taxa were shared between control and imidacloprid treatments, suggesting a degree of community stability, while certain taxa unique to each soil texture emerged as particularly sensitive or tolerant. Co‐occurrence network analysis further demonstrated that imidacloprid was associated with changes in bacterial association patterns, resulting in less connected and more modular networks, characterised by reduced complexity and increased modularity, particularly in clay and loamy sand soils. In parallel, functional pathway prediction indicated a community‐wide stress response, with upregulation of stress‐related pathways, such as amino acid degradation and membrane biosynthesis, and concurrent downregulation of pathways involved in energy and nutrient metabolism, including carbohydrate and nucleotide degradation. Taken together, these findings illustrate that while overall taxonomic richness remained relatively stable, imidacloprid exposure substantially reorganised bacterial interactions and functional capacities under the applied conditions, a reorganisation that may diminish soil microbial resilience, decelerate nutrient cycling and ultimately impair long‐term soil health. We emphasise, however, that the effects of imidacloprid exposure on soil bacterial communities were dependent on soil texture, which exerted a stronger influence on diversity, structure and function.

## Author Contributions

**Nilantha R. Hulugalle:** conceptualization, methodology, supervision, writing – review and editing. **Sharmin Akter:** conceptualization, methodology, data curation, investigation, validation, formal analysis, visualization, project administration, writing – original draft, writing – review and editing. **Craig L. Strong:** conceptualization, methodology, supervision, project administration, writing – review and editing. **Julia Jasonsmith:** conceptualization, methodology, supervision, writing – review and editing. **James O. Latimer:** resources, writing – review and editing.

## Funding

This work was supported by the Australian National University, the ANU University Research Scholarship (International) and the ANU HDR Fee Remission Merit Scholarship.

## Conflicts of Interest

The authors declare no conflicts of interest.

## Supporting information


**Table S1:** Analytical methods used to determine soil physicochemical properties.
**Table S2:** Summary of sequencing depth across treatment, soil, and sampling day groups.
**Table S3:** Analysis of deviance results for Chao1 richness estimate.
**Table S4:** Analysis of deviance results for Pielou's evenness.
**Table S5:** Analysis of deviance results for Faith's phylogenetic diversity (Faith's PD).
**Table S6:** Analysis of deviance results for Mean pairwise distance (MPD).
**Table S7:** Indicator taxa at the Order level across control and imidacloprid treatments in different soil types. Orders present in both treatments may represent distinct indicator ASVs differing at lower taxonomic ranks.
**Table S8:** Taxon names corresponding to the nodes of the co‐occurrence network for loamy sand soil, showing the treatments (control and imidacloprid).
**Table S9:** Taxon names corresponding to the nodes of the co‐occurrence network for sandy loam soil, showing the treatments (control and imidacloprid).
**Table S10:** Taxon names corresponding to the nodes of the co‐occurrence network for clay soil, showing the treatments (control and imidacloprid).
**Figure S1:** Rarefaction curve showing sequencing depth per sample.
**Figure S2:** Changes in phylogenetic diversity of bacterial communities across soil textures and treatments over time. (a) Faith's phylogenetic diversity (PD) and (b) mean pairwise distance (MPD). Points represent estimated marginal means ± SE (*n* = 3).

## Data Availability

All raw sequencing data have been submitted to the NCBI Sequence Read Archive under the accession number PRJNA1251260. Supporting datasets and materials necessary to interpret the findings are included in the main manuscript and Supporting Information.
