# Supplementary material for: Texture and Neonicotinoid Exposure Shape Bacterial Assemblages and Functions in Agricultural Soils: Responses Over Prolonged Exposure
Source: Environ Microbiol Rep. 2026 Aug 3;18(4):e70395. doi: 10.1111/1758-2229.70395 (PMC13433008; doi:10.1111/1758-2229.70395)
Supplement: Supplementary file 1 — Table S1: Analytical methods used to determine soil physicochemical properties. Table S2: Summary of sequencing depth across treatment, soil, and sampling day groups. Table S3: Analysis of deviance results for Chao1 richness estimate. Table S4: Analysis of deviance results for Pielou's evenness. Table S5: Analysis of deviance results for Faith's phylogenetic diversity (Faith's PD). Table S6: Analysis of deviance results for Mean pairwise distance (MPD). Table S7: Indicator taxa at the Order level across control and imidacloprid treatments in different soil types. Orders present in both treatments may represent distinct indicator ASVs differing at lower taxonomic ranks. Table S8: Taxon names corresponding to the nodes of the co‐occurrence network for loamy sand soil, showing the treatments (control and imidacloprid). Table S9: Taxon names corresponding to the nodes of the co‐occurrence network for sandy loam soil, showing the treatments (control and imidacloprid). Table S10: Taxon names corresponding to the nodes of the co‐occurrence network for clay soil, showing the treatments (control and imidacloprid). Figure S1: Rarefaction curve showing sequencing depth per sample. Figure S2: Changes in phylogenetic diversity of bacterial communities across soil textures and treatments over time. (a) Faith's phylogenetic diversity (PD) and (b) mean pairwise distance (MPD). Points represent estimated marginal means ± SE (n = 3). [file EMI4-18-e70395-s001.docx]

**Texture and neonicotinoid exposure shape bacterial assemblages and functions in agricultural soils: Responses over prolonged exposure**

Sharmin Akter*^1, 2^, Julia Jasonsmith^1^, Nilantha R. Hulugalle^1^, Craig L. Strong^1^, James O. Latimer^1^

^1^Fenner School of Environment and Society, College of Systems and Society, Australian National University, Canberra, ACT, Australia.

^2^Soil Resource Development Institute, Ministry of Agriculture, Dhaka, Bangladesh.

*** Corresponding author:**

Sharmin Akter, Fenner School of Environment and Society, Australian National University, Australia.

Email: [sharmin.akter@anu.edu.au](mailto:sharmin.akter@anu.edu.au)

ORCID: 0000-0002-4086-3439

**List of Supplementary tables:**

**Table S1:** Analytical methods used to determine soil physicochemical properties

**Table S2:** Summary of sequencing depth across treatment, soil, and sampling day groups

**Table S3:** Analysis of deviance results for Chao1 richness estimate

**Table S4:** Analysis of deviance results for Pielou’s evenness

**Table S5:** Analysis of deviance results for Faith’s phylogenetic diversity (Faith’s PD)

**Table S6:** Analysis of deviance results for Mean pairwise distance (MPD)

**Table S7:** Indicator taxa at the Order level across control and imidacloprid treatments in different soil types. Orders present in both treatments may represent distinct indicator ASVs differing at lower taxonomic ranks

**Table S8:** Taxon names corresponding to the nodes of the co-occurrence network for loamy sand soil, showing the treatments (control and imidacloprid)

**Table S9:** Taxon names corresponding to the nodes of the co-occurrence network for sandy loam soil, showing the treatments (control and imidacloprid)

**Table S10:** Taxon names corresponding to the nodes of the co-occurrence network for clay soil, showing the treatments (control and imidacloprid)

**List of Supplementary figures:**

**Figure S1.** Rarefaction curve showing sequencing depth per sample.

**Figure S2.** Changes in phylogenetic diversity of bacterial communities across soil textures and treatments over time. (a) Faith’s phylogenetic diversity (PD) and (b) Mean Pairwise Distance (MPD). Points represent estimated marginal means ± SE (n = 3).

**Table S1:** Analytical methods used to determine soil physicochemical properties

| Parameter | Method | Unit | Method reference |
| --- | --- | --- | --- |
| Texture | USDA textural triangle | - | Soil Science Division Staff (2017) |
| Clay | Hydrometer method | g/100 g | (modified) Carter and Gregorich (2007) |
| Silt | Hydrometer method | g/100 g |  |
| Sand | Hydrometer method | g/100 g |  |
| pH | 1:5 soil:water suspension | - | Rayment and Lyons (2011) |
| Electrical conductivity | 1:5 soil:water extract | dS/m |  |
| Organic matter | Calculation: Total Carbon × 1.75 | g/100 g |  |
| Effective Cation Exchange Capacity | A sum of Ca, Mg, K, Na | - |  |
| Exchangeable Ca | Ammonium Acetate (1M NH_4_OAc) | cmol_+_/kg |  |
| Exchangeable Mg |  | cmol_+_/kg |  |
| Exchangeable K |  | cmol_+_/kg |  |
| Exchangeable Sodium Percentage | Base Saturation Calculations -  Cation cmol+/kg / ECEC × 100 | % |  |
| Ca:Mg ratio | Calculation: Ca/ Mg | - |  |
| Available Phosphorus | Fluoride-extractable P (Bray 1-P) | Mg P/kg |  |
| Total Carbon | LECO TruMac Analyzer | g/100 g | LECO Corporation (2015) |
| Total Nitrogen | LECO TruMac Analyzer | g/100 g |  |
| C:N ratio | Calculation:  Total Carbon/ Total Nitrogen | - |  |

**Table S2:** Summary of sequencing depth across treatment, soil, and sampling day groups

| Treatment | Soil | Day | n | Mean | SD | Min | Max |
| --- | --- | --- | --- | --- | --- | --- | --- |
| Control | Loamy sand | 3 | 3 | 5147 | 3246 | 2235 | 8646 |
|  |  | 7 |  | 5608 | 2370 | 2874 | 7086 |
|  |  | 14 |  | 8905 | 2746 | 6499 | 11896 |
|  |  | 21 |  | 8113 | 2175 | 6053 | 10387 |
|  |  | 28 |  | 7064 | 274 | 6752 | 7264 |
|  | Sandy loam | 3 |  | 6969 | 2260 | 5360 | 9553 |
|  |  | 7 |  | 8306 | 803 | 7662 | 9205 |
|  |  | 14 |  | 8180 | 1328 | 7069 | 9651 |
|  |  | 21 |  | 8190 | 4119 | 3469 | 11054 |
|  |  | 28 |  | 6521 | 449 | 6011 | 6854 |
|  | Clay | 3 |  | 7155 | 1657 | 6051 | 9060 |
|  |  | 7 |  | 6942 | 2295 | 4461 | 8989 |
|  |  | 14 |  | 6386 | 1324 | 5191 | 7810 |
|  |  | 21 |  | 8962 | 1605 | 7480 | 10666 |
|  |  | 28 |  | 3588 | 1350 | 2036 | 4484 |
| Imidacloprid | Loamy sand | 3 |  | 5716 | 4183 | 1274 | 9579 |
|  |  | 7 |  | 6285 | 1249 | 4924 | 7380 |
|  |  | 14 |  | 8417 | 3114 | 4933 | 10930 |
|  |  | 21 |  | 8883 | 812 | 7963 | 9501 |
|  |  | 28 |  | 6879 | 1234 | 5465 | 7740 |
|  | Sandy loam | 3 |  | 6815 | 2867 | 5128 | 10125 |
|  |  | 7 |  | 8494 | 3183 | 5559 | 11878 |
|  |  | 14 |  | 7244 | 3832 | 2819 | 9480 |
|  |  | 21 |  | 8480 | 3536 | 5047 | 12110 |
|  |  | 28 |  | 4723 | 650 | 4304 | 5472 |
|  | Clay | 3 |  | 6604 | 1849 | 4491 | 7925 |
|  |  | 7 |  | 6701 | 2529 | 3802 | 8453 |
|  |  | 14 |  | 6126 | 2782 | 3277 | 8836 |
|  |  | 21 |  | 5704 | 1429 | 4331 | 7183 |
|  |  | 28 |  | 4257 | 1336 | 2829 | 5476 |

**Table S3:** Analysis of deviance results for Chao1 richness estimate

| Index | Effect | Chi-square | Df | Pr(>Chisq) | Significance |
| --- | --- | --- | --- | --- | --- |
| Chao1 | Treatment | 1.8484 | 1 | 0.1740 |  |
|  | Soil | 32.2417 | 2 | 9.972e-08 | *** |
|  | Day | 5.9606 | 4 | 0.2021 |  |
|  | Treatment:Soil | 1.2108 | 2 | 0.5459 |  |
|  | Treatment:Day | 0.8935 | 4 | 0.9255 |  |
|  | Soil:Day | 8.3376 | 8 | 0.4012 |  |
|  | Treatment:Soil:Day | 3.6117 | 8 | 0.8903 |  |

*Signif. codes: 0 ‘***’ 0.001 ‘**’ 0.01 ‘*’ 0.05 ‘.’ 0.1 ‘ ’ 1*

**Table S4:** Analysis of deviance results for Pielou’s evenness

| Index | Effect | Chi-square | Df | Pr(>Chisq) | Significance |
| --- | --- | --- | --- | --- | --- |
| Pielou’s evenness | Treatment | 3.6856 | 1 | 0.0548857 | . |
|  | Soil | 4.2052 | 2 | 0.1221374 |  |
|  | Day | 19.0723 | 4 | 0.0007606 | *** |
|  | Treatment:Soil | 0.5427 | 2 | 0.7623347 |  |
|  | Treatment:Day | 3.6603 | 4 | 0.4539336 |  |
|  | Soil:Day | 7.4540 | 8 | 0.4885350 |  |
|  | Treatment:Soil:Day | 5.6453 | 8 | 0.6868890 |  |

*Signif. codes: 0 ‘***’ 0.001 ‘**’ 0.01 ‘*’ 0.05 ‘.’ 0.1 ‘ ’ 1*

**Table S5:** Analysis of deviance results for Faith’s phylogenetic diversity (Faith’s PD)

| Index | Effect | Chi-square | Df | Pr(>Chisq) | Significance |
| --- | --- | --- | --- | --- | --- |
| Faith’s PD | Treatment | 0.1137 | 1 | 0.7360 |  |
|  | Soil | 44.9658 | 2 | 1.721e-10 | *** |
|  | Day | 27.1688 | 4 | 1.838e-05 | *** |
|  | Treatment:Soil | 1.5978 | 2 | 0.4498 |  |
|  | Treatment:Day | 2.5758 | 4 | 0.6311 |  |
|  | Soil:Day | 12.0115 | 8 | 0.1507 |  |
|  | Treatment:Soil:Day | 4.7483 | 8 | 0.7841 |  |

*Signif. codes: 0 ‘***’ 0.001 ‘**’ 0.01 ‘*’ 0.05 ‘.’ 0.1 ‘ ’ 1*

**Table S6:** Analysis of deviance results for Mean pairwise distance (MPD)

| Index | Effect | Chi-square | Df | Pr(>Chisq) | Significance |
| --- | --- | --- | --- | --- | --- |
| MPD | Treatment | 3.7373 | 1 | 0.05321 | . |
|  | Soil | 47.4405 | 2 | 4.994e-11 | *** |
|  | Day | 10.4674 | 4 | 0.03325 | * |
|  | Treatment:Soil | 1.4224 | 2 | 0.49106 |  |
|  | Treatment:Day | 8.7640 | 4 | 0.06728 | . |
|  | Soil:Day | 7.9172 | 8 | 0.44160 |  |
|  | Treatment:Soil:Day | 6.4773 | 8 | 0.59392 |  |

*Signif. codes: 0 ‘***’ 0.001 ‘**’ 0.01 ‘*’ 0.05 ‘.’ 0.1 ‘ ’ 1*

**Table S7:** Indicator taxa at the Order level across control and imidacloprid treatments in different soil types. Orders present in both treatments may represent distinct indicator ASVs differing at lower taxonomic ranks

| Soil texture | Control indicator taxa (Order) | Imidacloprid indicator taxa (Order) |
| --- | --- | --- |
| Loamy sand | Rhizobiales, Pseudomonadales, Propionibacteriales, Cytophagales, Solirubrobacterales, Propionibacteriales, Vicinamibacterales | Polyangiales, Micrococcales, Corynebacteriales, Propionibacteriales, Rhizobiales, Flavobacteriales, Solirubrobacterales, Frankiales, Burkholderiales, Cytophagales, Corynebacteriales, Vicinamibacterales |
| Sandy loam | Rhizobiales, Gaiellales, Kineosporiales, Burkholderiales, Propionibacteriales, Solirubrobacterales, Cytophagales, Thermomicrobiales, Streptomycetales, Caulobacterales, Gemmatimonadales, Alicyclobacillales, Xanthomonadales, Corynebacteriales, Chitinophagales, Rhodobacterales, Micropepsales, Acetobacterales | Gammaproteobacteria Incertae Sedis, Burkholderiales, Cytophagales, Propionibacteriales, Streptomycetales, Pseudonocardiales, Acetobacterales, Rhizobiales, Pseudomonadales, Chitinophagales, Flavobacteriales |
| Clay | Streptomycetales, Gemmatimonadales, Solirubrobacterales, Burkholderiales, Pseudonocardiales, Frankiales, Gaiellales, Bacillales, Thermomicrobiales, Vicinamibacterales, Cytophagales, Propionibacteriales | Rhizobiales |

**Table S8:** Taxon names corresponding to the nodes of the co-occurrence network for loamy sand soil, showing the treatments (control and imidacloprid)

| Control | | Imidacloprid | |
| --- | --- | --- | --- |
| Node number | Taxon (Order) | Node number | Taxon (Order) |
| 1 | Azospirillales | 1 | Acetobacterales |
| 2 | Blastocatellales | 2 | Acholeplasmatales |
| 3 | Chitinophagales | 3 | Alicyclobacillales |
| 4 | Chthoniobacterales | 4 | Bacillales |
| 5 | Corynebacteriales | 5 | Corynebacteriales |
| 6 | Paenibacillales | 6 | Desulfotomaculales |
| 7 | Acetobacterales | 7 | Frankiales |
| 8 | Acidimicrobiales | 8 | Gemmatales |
| 9 | Acidobacteriales | 9 | Micromonosporales |
| 10 | Alicyclobacillales | 10 | Propionibacteriales |
| 11 | Babeliales | 11 | Solirubrobacterales |
| 12 | Bacillales | 12 | Streptomycetales |
| 13 | Diplorickettsiales | 13 | Acidobacteriales |
| 14 | Fibrobacterales | 14 | Bdellovibrionales |
| 15 | Frankiales | 15 | Bryobacterales |
| 16 | Gaiellales | 16 | Dongiales |
| 17 | Gammaproteobacteria Incertae Sedis | 17 | Gaiellales |
| 18 | Gemmatales | 18 | Gammaproteobacteria Incertae Sedis |
| 19 | Gemmatimonadales | 19 | Gemmatimonadales |
| 20 | Haliangiales | 20 | Kineosporiales |
| 21 | Kineosporiales | 21 | Nitrospirales |
| 22 | Micrococcales | 22 | Polyangiales |
| 23 | Micromonosporales | 23 | Pseudonocardiales |
| 24 | Micropepsales | 24 | Reyranellales |
| 25 | Nitrospirales | 25 | Steroidobacterales |
| 26 | Pedosphaerales | 26 | Thermoanaerobaculales |
| 27 | Pirellulales | 27 | Acidiferrobacterales |
| 28 | Polyangiales | 28 | Dethiobacterales |
| 29 | Propionibacteriales | 29 | Azospirillales |
| 30 | Reyranellales | 30 | Burkholderiales |
| 31 | Rhodobacterales | 31 | Chitinophagales |
| 32 | Solirubrobacterales | 32 | Cytophagales |
| 33 | Sphingomonadales | 33 | Flavobacteriales |
| 34 | Streptomycetales | 34 | Pseudomonadales |
| 35 | Streptosporangiales | 35 | Elsterales |
| 36 | Thermoanaerobaculales | 36 | Kallotenuales |
| 37 | Thermomicrobiales | 37 | Fibrobacterales |
| 38 | Kallotenuales | 38 | Myxococcales |
| 39 | Obscuribacterales | 39 | Oscillospirales |
| 40 | Rickettsiales | 40 | Thermincolales |
| 41 | Silvanigrellales | 41 | Micrococcales |
| 42 | Burkholderiales | 42 | Vicinamibacterales |
| 43 | Cytophagales | 43 | Sphingomonadales |
| 44 | Flavobacteriales | 44 | Haliangiales |
| 45 | Pseudomonadales | 45 | Streptosporangiales |
| 46 | Micavibrionales | 46 | Longimicrobiales |
| 47 | Desulfitibacterales | 47 | Ectothiorhodospirales |
| 48 | Dethiobacterales | 48 | Verrucomicrobiales |
| 49 | Rokubacteriales | 49 | Saccharimonadales |
| 50 | Caldilineales | 50 | Rhodospirillales |
| 51 | Rhizobiales | 51 | Rhizobiales |
| 52 | Xanthomonadales | 52 | Veillonellales-Selenomonadales |
| 53 | Sphingobacteriales |  |  |
| 54 | Pseudonocardiales |  |  |
| 55 | Vicinamibacterales |  |  |
| 56 | Tepidisphaerales |  |  |
| 57 | Steroidobacterales |  |  |
| 58 | Veillonellales-Selenomonadales |  |  |
| 59 | Verrucomicrobiales |  |  |
| 60 | Sumerlaeales |  |  |
| 61 | Hydrogenedentiales |  |  |
| 62 | Opitutales |  |  |
| 63 | Thermincolales |  |  |

**Table S9:** Taxon names corresponding to the nodes of the co-occurrence network for sandy loam soil, showing the treatments (control and imidacloprid)

| Control | | Imidacloprid | |
| --- | --- | --- | --- |
| Node number | Taxon (Order) | Node number | Taxon (Order) |
| 1 | Azospirillales | 1 | Acetobacterales |
| 2 | Blastocatellales | 2 | Alicyclobacillales |
| 3 | Chitinophagales | 3 | Bacillales |
| 4 | Chthoniobacterales | 4 | Caldilineales |
| 5 | Corynebacteriales | 5 | Caulobacterales |
| 6 | Rhizobiales | 6 | Corynebacteriales |
| 7 | Acetobacterales | 7 | Frankiales |
| 8 | Alicyclobacillales | 8 | Micromonosporales |
| 9 | Babeliales | 9 | Propionibacteriales |
| 10 | Bacillales | 10 | Solirubrobacterales |
| 11 | Bdellovibrionales | 11 | Bdellovibrionales |
| 12 | Diplorickettsiales | 12 | Dongiales |
| 13 | Frankiales | 13 | Gaiellales |
| 14 | Gaiellales | 14 | Gammaproteobacteria Incertae Sedis |
| 15 | Gammaproteobacteria Incertae Sedis | 15 | Gemmatimonadales |
| 16 | Gemmatimonadales | 16 | Nitrospirales |
| 17 | Haliangiales | 17 | Polyangiales |
| 18 | Micrococcales | 18 | Reyranellales |
| 19 | Micromonosporales | 19 | Steroidobacterales |
| 20 | Myxococcales | 20 | Chthoniobacterales |
| 21 | Nitrospirales | 21 | Lachnospirales |
| 22 | Pirellulales | 22 | Rhizobiales |
| 23 | Polyangiales | 23 | Fimbriimonadales |
| 24 | Propionibacteriales | 24 | Haliangiales |
| 25 | Pseudonocardiales | 25 | Micrococcales |
| 26 | Reyranellales | 26 | Rhodobacterales |
| 27 | Rhodobacterales | 27 | Thermomicrobiales |
| 28 | Solirubrobacterales | 28 | Burkholderiales |
| 29 | Steroidobacterales | 29 | Chitinophagales |
| 30 | Streptosporangiales | 30 | Clostridiales |
| 31 | Caldilineales | 31 | Flavobacteriales |
| 32 | Anaerolineales | 32 | Paenibacillales |
| 33 | Burkholderiales | 33 | Pseudomonadales |
| 34 | Fimbriimonadales | 34 | Bacteriovoracales |
| 35 | Flavobacteriales | 35 | Rhodospirillales |
| 36 | Micavibrionales | 36 | Azospirillales |
| 37 | Acidiferrobacterales | 37 | Verrucomicrobiales |
| 38 | Kiloniellales | 38 | Pirellulales |
| 39 | Nitrococcales | 39 | Enterobacterales |
| 40 | Fibrobacterales | 40 | Vicinamibacterales |
| 41 | Paenibacillales | 41 | Planctomycetales |
| 42 | Vicinamibacterales | 42 | Xanthomonadales |
| 43 | Microtrichales | 43 | Streptomycetales |
| 44 | Gemmatales | 44 | Pseudonocardiales |
| 45 | Streptomycetales | 45 | Sphingomonadales |
| 46 | Thermomicrobiales | 46 | Microtrichales |
| 47 | Planctomycetales | 47 | Tepidisphaerales |
| 48 | Cytophagales | 48 | Myxococcales |
| 49 | Pseudomonadales | 49 | Sphingobacteriales |
| 50 | Isosphaerales | 50 | Oligoflexales |
| 51 | Spirochaetales | 51 | Cytophagales |
| 52 | Symbiobacteriales | 52 | Salinisphaerales |
| 53 | Vampirovibrionales | 53 | Rokubacteriales |
|  |  | 54 | Rickettsiales |

**Table S10:** Taxon names corresponding to the nodes of the co-occurrence network for clay soil, showing the treatments (control and imidacloprid)

| Control | | Imidacloprid | |
| --- | --- | --- | --- |
| Node number | Taxon (Order) | Node number | Taxon (Order) |
| 1 | Azospirillales | 1 | Alicyclobacillales |
| 2 | Chitinophagales | 2 | Bacillales |
| 3 | Rhizobiales | 3 | Desulfotomaculales |
| 4 | Babeliales | 4 | Gemmatales |
| 5 | Bacillales | 5 | Micromonosporales |
| 6 | Frankiales | 6 | Obscuribacterales |
| 7 | Gaiellales | 7 | Propionibacteriales |
| 8 | Gemmatimonadales | 8 | Solirubrobacterales |
| 9 | Glycomycetales | 9 | Bryobacterales |
| 10 | Haliangiales | 10 | Gaiellales |
| 11 | Micrococcales | 11 | Gemmatimonadales |
| 12 | Nitrospirales | 12 | Nitrospirales |
| 13 | Pirellulales | 13 | Rubrobacterales |
| 14 | Propionibacteriales | 14 | Rhizobiales |
| 15 | Pyrinomonadales | 15 | Sphingomonadales |
| 16 | Reyranellales | 16 | Symbiobacteriales |
| 17 | Rhodobacterales | 17 | Burkholderiales |
| 18 | Tepidisphaerales | 18 | Aneurinibacillales |
| 19 | Burkholderiales | 19 | Bacteriovoracales |
| 20 | Cytophagales | 20 | Glycomycetales |
| 21 | Thermomicrobiales | 21 | Ardenticatenales |
| 22 | Vicinamibacterales | 22 | Vicinamibacterales |
| 23 | Anaerolineales | 23 | Cytophagales |
| 24 | Isosphaerales | 24 | Fibrobacterales |
| 25 | Xanthomonadales | 25 | Rhodobacterales |
| 26 | Tistrellales | 26 | Steroidobacterales |
| 27 | Planctomycetales | 27 | Salinisphaerales |
| 28 | Bacteroidales | 28 | Pseudonocardiales |
| 29 | Micromonosporales | 29 | Xanthomonadales |
| 30 | Salinisphaerales | 30 | Diplorickettsiales |
| 31 | Polyangiales | 31 | Polyangiales |
| 32 | Solirubrobacterales | 32 | Thermomicrobiales |
| 33 | Steroidobacterales | 33 | Syntrophobacterales |
| 34 | Sphingobacteriales | 34 | Erysipelotrichales |
| 35 | Thermoactinomycetales | 35 | Pedosphaerales |

**
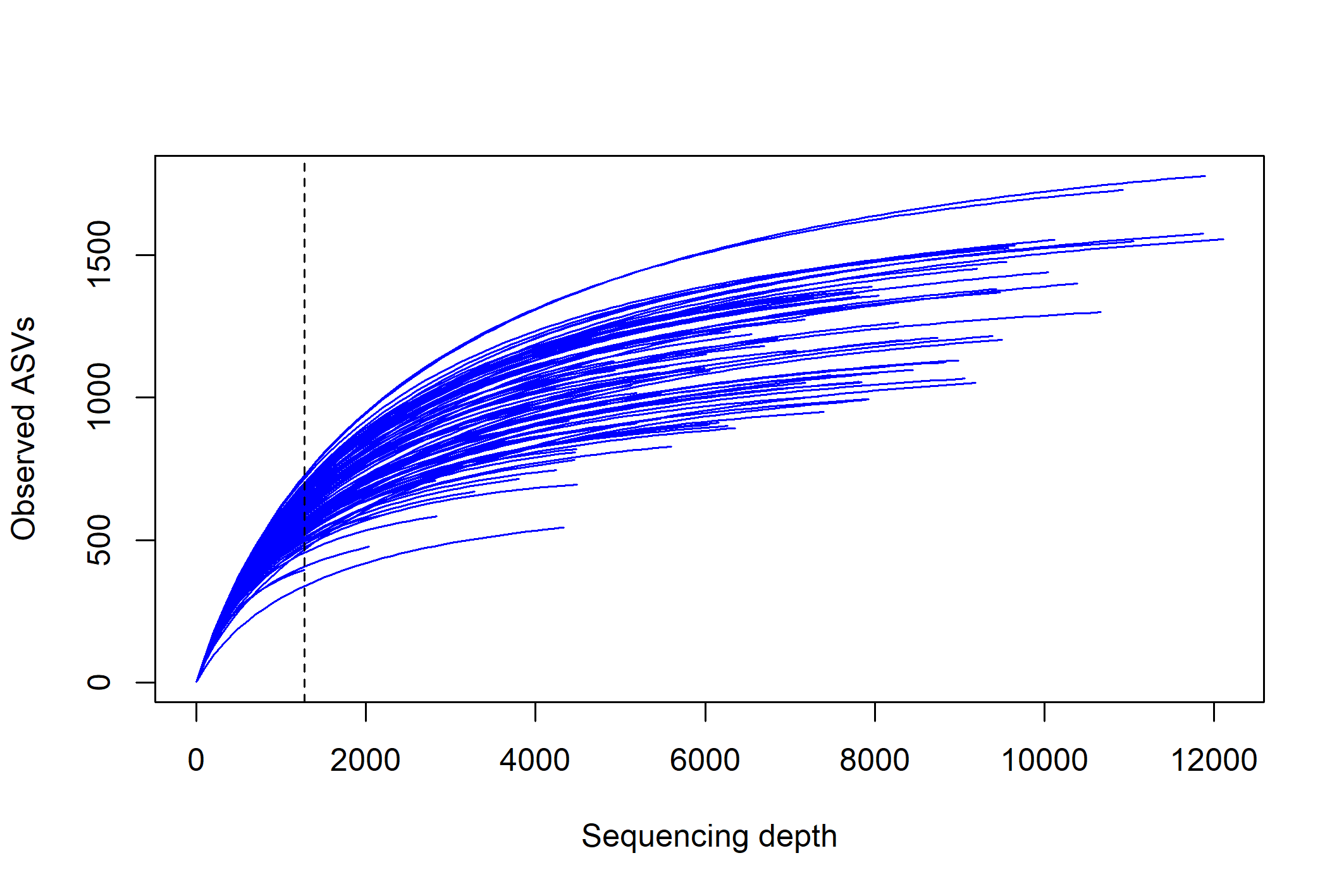
**

**Figure S1.** Rarefaction curve showing sequencing depth per sample.

**
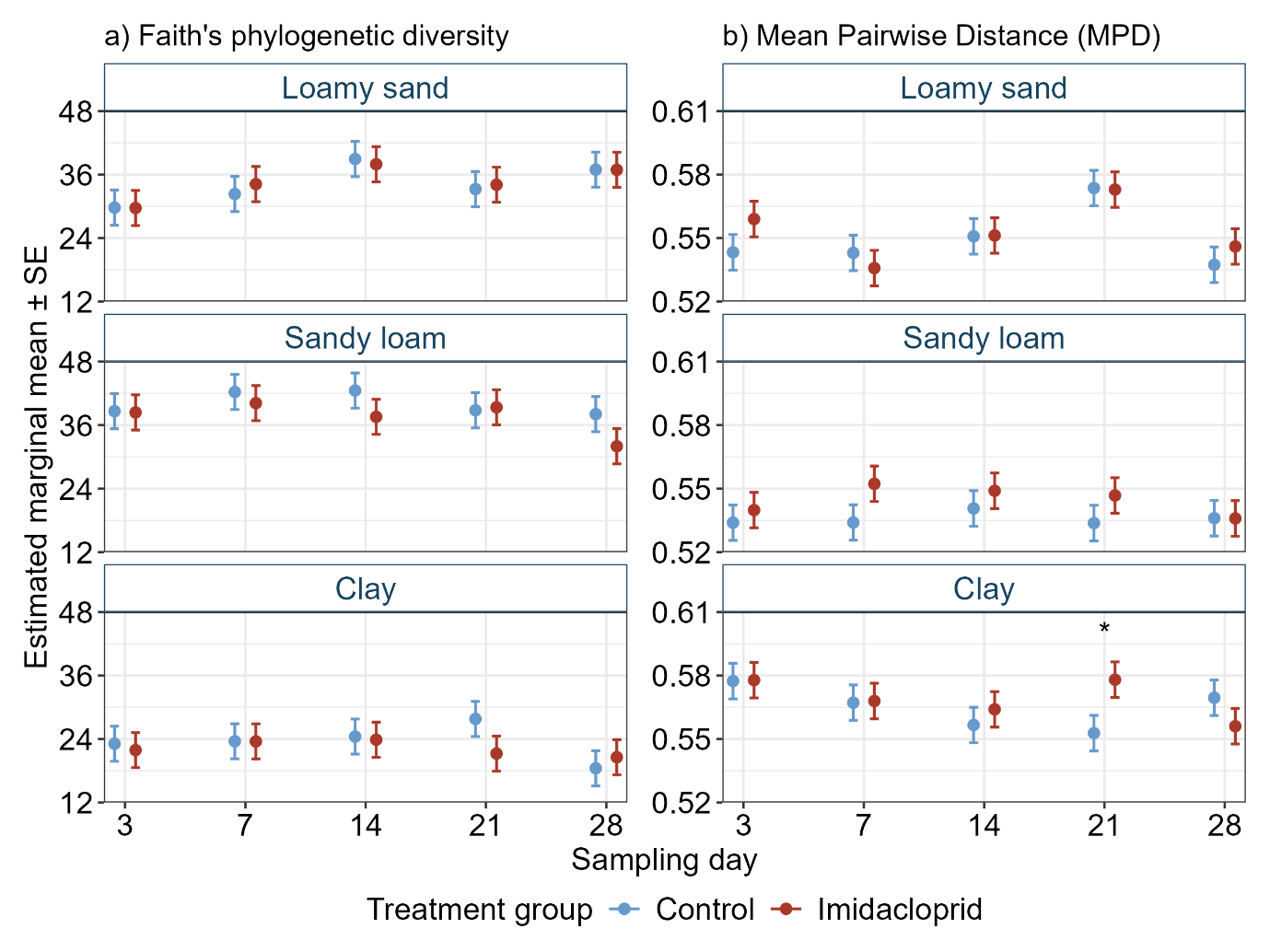
**

**Figure S2.** Changes in phylogenetic diversity of bacterial communities across soil textures and treatments over time. (a) Faith’s phylogenetic diversity (PD) and (b) Mean Pairwise Distance (MPD). Points represent estimated marginal means ± SE (n = 3).

**References**

Carter, M. R., & Gregorich, E. G. (2007). *Soil sampling and methods of analysis* (Second ed.). CRC Press. <https://doi.org/10.1201/9781420005271>

LECO Corporation. (2015). *Carbon, nitrogen, and sulfur in soil (Instrument: TruMac® CNS)*. Retrieved 14 April 2025 from <https://lecocorp.ams3.digitaloceanspaces.com/wp-content/uploads/20240102152934/trumac_cns_soil_203-821-498.pdf>

Rayment, G. E., & Lyons, D. J. (2011). *Soil chemical methods - Australasia*. CSIRO PUBLISHING. <https://doi.org/10.1071/9780643101364>

Soil Science Division Staff. (2017). Examination and description of soil profiles. In C. Ditzler, K. Scheffe, & H. C. Monger (Eds.), *Soil survey manual* (4th ed., pp. 83-233). USDA Handbook 18, Government Printing Office, Washington, D.C. <https://www.nrcs.usda.gov/resources/guides-and-instructions/soil-survey-manual>
